# Tertiary Hyperparathyroidism: A Case Report

**DOI:** 10.7759/cureus.70179

**Published:** 2024-09-25

**Authors:** Carolina Fernandes, Vera Vieira, Cláudia Diogo, Ana Catarina Domingues, Ana Rodrigues

**Affiliations:** 1 Internal Medicine Department, Unidade Local de Saúde da Região de Leiria, Leiria, PRT; 2 Intensive Care Unit, Unidade Local de Saúde da Região de Leiria, Leiria, PRT

**Keywords:** adenoma, bariatric surgery, hypercalcemia, malabsorption, tertiary hyperparathyroidism

## Abstract

Tertiary hyperparathyroidism is characterized by increased parathyroid hormone (PTH) secretion that appears after prolonged secondary hyperparathyroidism, leading to the onset of hypercalcemia. The parathyroid glands are found to undergo hyperplastic or adenomatous changes and act autonomously with increased parathormone secretion not suppressed by feedback control. This entity is commonly associated with long-term secondary hyperparathyroidism states, such as chronic kidney disease, renal transplantation, and gastrointestinal malabsorption.

The authors describe the case of a 50-year-old female with a history of biliopancreatic diversion surgery, renal lithiasis with recurrent obstructive pyelonephritis, and a work accident with significant limitation of mobility. She was referred to hospital consultation for a pathological fracture of the dorsal vertebra in the context of tertiary hyperparathyroidism due to long-term gastrointestinal malabsorption.

## Introduction

The main role of parathyroid hormone (PTH) is the regulation of serum calcium levels. Low levels of calcium and vitamin D stimulate the secretion of this hormone. PTH leads to an increase in calcium levels through its resorption on renal tubules, the resorption of calcium and phosphate on bone tissue, and the stimulation of calcitriol synthesis with consequent calcium absorption in the intestine [1].

Tertiary hyperparathyroidism is characterized by an exaggerated secretion of PTH due to prolonged secondary hyperparathyroidism, leading to the development of hypercalcemia. The parathyroid glands act autonomously with PTH secretion not suppressed through negative feedback [2]. This entity is commonly associated with chronic kidney disease, but it can be related to any disorder that leads to secondary hyperparathyroidism with long-lasting hypercalcemia, for example, gastrointestinal malabsorption [3].

The signs and symptoms of tertiary hyperparathyroidism are the same as primary hyperparathyroidism, bone pain, decreased bone mineral density, osteitis fibrosa cystica with pathological fractures, pruritus, muscular weakness, nephrolithiasis, anorexia, nausea, and weight loss, and they result from increased PTH levels and/or hypercalcemia [2].

The authors describe a clinical report about tertiary hyperparathyroidism due to gastrointestinal malabsorption secondary to bariatric surgery.

## Case presentation

This case describes a 50-year-old female with a past medical history of morbid obesity who underwent a biliopancreatic diversion procedure at the age of 38 years old, nephrolithiasis with recurrent pyelonephritis, and a work accident with a consequent motor impairment that led her to be bedridden. The patient was lost to follow-up after bariatric surgery and had no screening for possible micronutrient deficiencies or was not under any supplementation.

She was referred to hospital consultation due to a pathological fracture of the 11th dorsal vertebra. The patient complained of lumbar pain that begun three months before and was associated with anorexia with weight loss and generalized muscle weakness with worsening of motor impairment.

Upon examination, the patient had an emaciated appearance, heart and lung sounds were normal, and no adenopathies or neck lumps were identified. She presented with pain throughout her dorsolumbar spine.

The first diagnostic hypothesis was bone metastasis with an unknown primary tumor. The patient was submitted to several examinations such as body computed tomography, upper and lower endoscopies, and mammography to exclude any possible primary tumors. A positron emission tomography (PET) was done with no areas suggestive of tumor lesions. After that, the authors investigated other possible causes that could lead to the development of the bone lesions described.

From the laboratory evaluation (Table 1), the following stand out: mild hypercalcemia (calcium correction for albumin of 2.8 mmol/L), hypophosphatemia (0.58 mmol/L), a significant increase of alkaline phosphatase (2176 U/L) and PTH (2410 pg/mL), and vitamin D deficiency (6.94 ng/mL).

**Table 1:** Blood investigation PTH: parathyroid hormone

Blood analysis	Results	Reference range
Calcium	2.45 mmol/L	2.20-2.65 mmol/L
Phosphate	0.58 mmol/L	0.81-1.45 mmol/L
Magnesium	0.62 mmol/L	0.77-1.03 mmol/L
Albumin	24 g/L	35-52 g/L
Alkaline phosphatase	2176 U/L	30-120 U/L
PTH	2410.5 pg/mL	12.0-88.0 pg/mL
Vitamin D	6.94 ng/mL	30-100 ng/mL

Computed tomography showed the kidneys with normal size and morphology with bilateral urinary tract dilation and nonobstructive calculi on both kidneys and ureters (the biggest one with 9×11 mm). There were no adenopathies or suspicious nodules identified.

**Figure 1:**
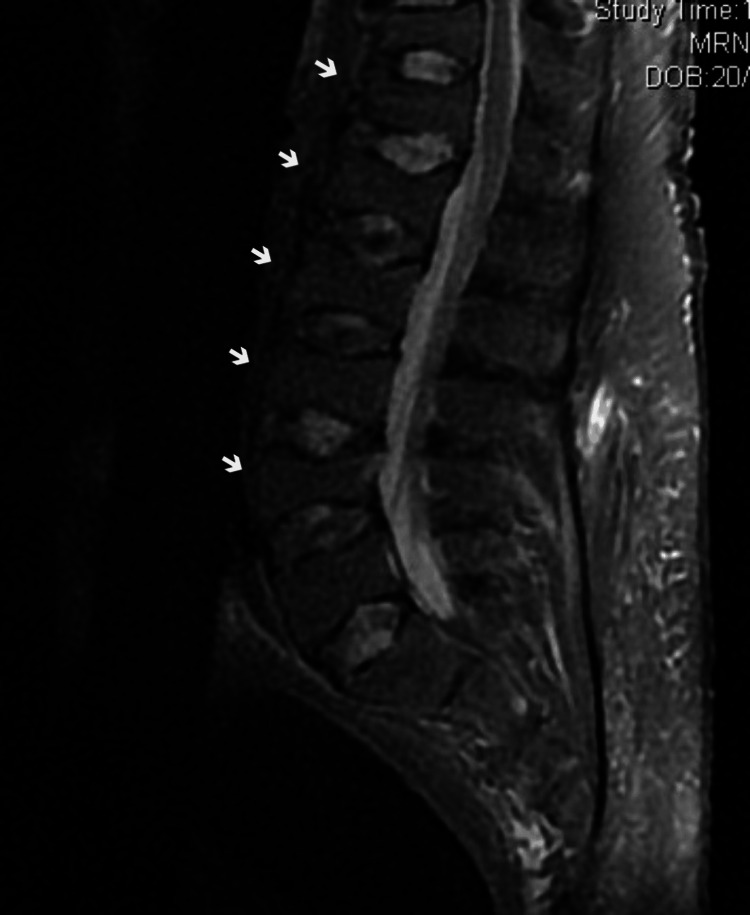
Sagittal view of dorsolumbar magnetic resonance imaging Arrows: compressive pathological fractures

Dorsolumbar magnetic resonance imaging (Figure 1) showed the diffused variation of the normal signal emission of the bone marrow compatible with bone resorption and multiple compressive pathological fractures throughout the lumbar spine and dorsolumbar transition.

The neck ultrasound did not identify any nodules in the thyroid or parathyroid glands.

**Figure 2:**
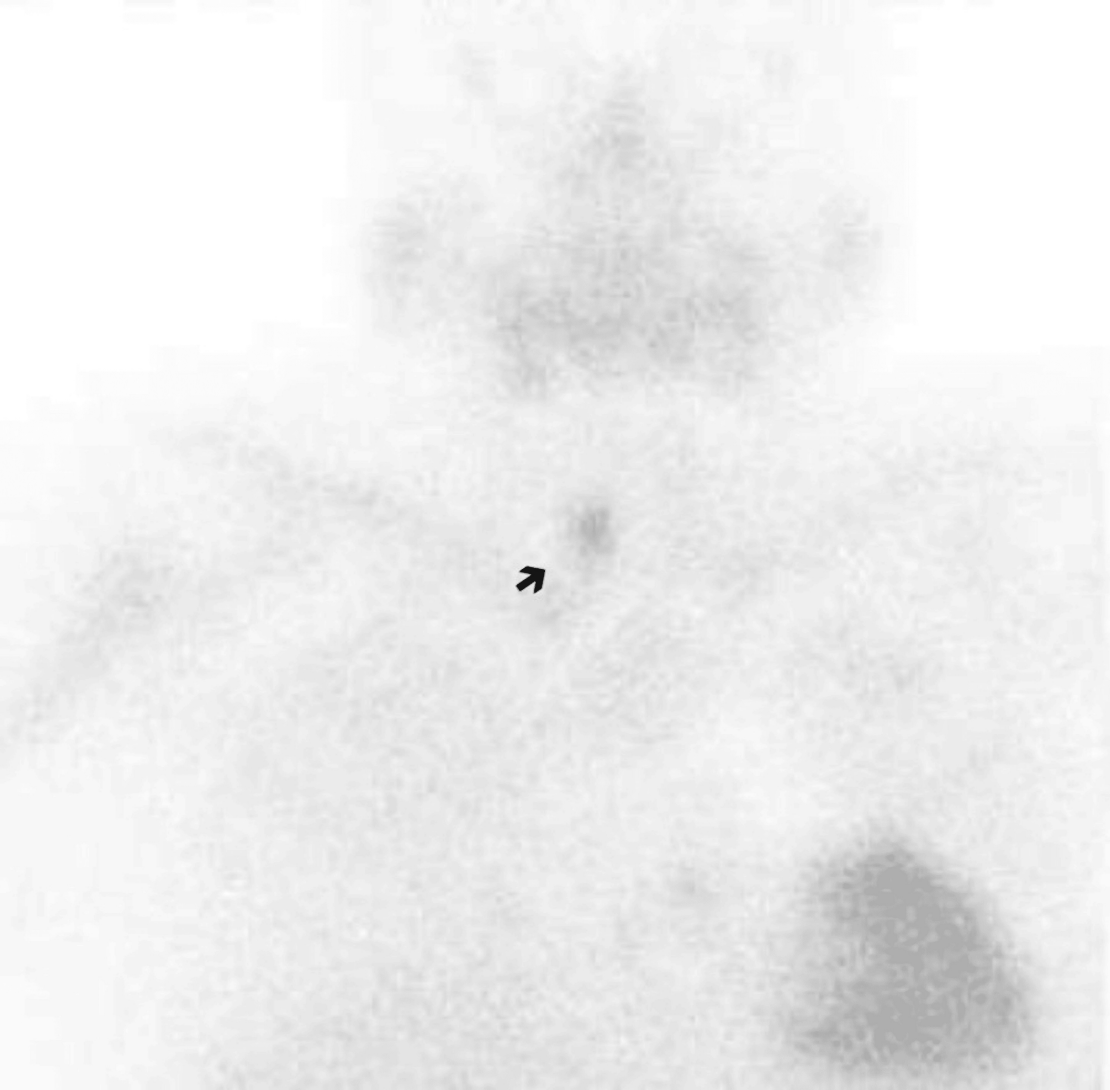
Parathyroid gland scintigraphy Arrow: right inferior parathyroid gland adenoma

The patient was also submitted to parathyroid gland scintigraphy (Figure 2), which showed radionuclide accumulation compatible with an adenoma in the right inferior parathyroid gland.

After the revision of the clinical case, tertiary hyperparathyroidism was established as a diagnostic hypothesis. The patient was referred for the surgical removal of the parathyroid adenoma identified.

While she was waiting for the surgery, the patient developed obstructive pyelonephritis with septic shock and was admitted to the intensive care unit but unfortunately died.

## Discussion

The analysis of this clinical case has several special considerations.

First, the patient suffered from a malabsorption syndrome secondary to bariatric surgery with a severe vitamin D deficiency. The fact that she was bedridden with nearly no sun exposure contributed to the worsening of hypovitaminosis D. Micronutrient deficiencies are common in the majority of bariatric procedures, and the most frequent are of calcium, vitamin D, iron, vitamin B12, folic acid, copper, and zinc. The patient was submitted to a biliopancreatic diversion, which led to a bypass of the main area of calcium absorption in the intestine and an increased risk of developing hypocalcemia and secondary hyperparathyroidism [4].

Second, the PTH serum levels were extremely high, which usually does not occur in secondary hyperparathyroidism. Furthermore, the parathyroid gland scintigraphy showed radionuclide accumulation compatible with a parathyroid gland adenoma that is compatible with tertiary hyperparathyroidism. Both secondary and tertiary hyperparathyroidism result from a chronic stimulus to PTH secretion, but the serum calcium is always normal in the former, whereas it is always elevated in the latter [2]. The patient had only mild hypercalcemia that was due to the malabsorption syndrome related to bariatric surgery and hypovitaminosis D.

Third, it would be important to exclude parathyroid carcinoma. Parathyroid carcinoma is extremely rare with an incidence of 1.25 per 10 million patients. Usually, it presents as a palpable cervical lump with hard consistency and adherent to the deep tissues with a median size of 3 cm. It is also associated with extremely high levels of serum PTH and hypercalcemia [5]. At the time of parathyroid carcinoma diagnosis, it is common that the tumor is of increased size with bone and renal lesions and with significant hypercalcemia when compared to parathyroid adenoma [6]. The patient was submitted to PET, which also contributes to excluding this hypothesis.

## Conclusions

Tertiary hyperparathyroidism is a rare condition that can be associated with devastating complications for the patient. Its diagnosis demands the combination of a thorough medical history with diagnostic tests including blood work and bone and parathyroid gland imaging.

This case report describes a serious complication of nutritional deficiency after bariatric surgery that developed several years after the procedure. This highlights the importance of the follow-up after bariatric surgery with a lifelong screening of micronutrients and their supplementation to avoid life-threatening complications.
